# MicroRNA-mediated autophagy regulation in thyroid cancer drug resistance

**DOI:** 10.20517/cdr.2025.73

**Published:** 2025-06-18

**Authors:** Dongye Huang, Qianwen Liu, Chang Liu, Jingna Cao, Senmin Zhang, Huijiao Cao, Wenkuan Chen

**Affiliations:** ^1^State Key Laboratory of Oncology in South China, Guangdong Provincial Clinical Research Center for Cancer, Sun Yat-sen University Cancer Center, Guangzhou 510060, Guangdong, China.; ^2^Department of Endocrinology and Metabolism, The First People’s Hospital of Chenzhou, The First School of Clinical Medicine, University of Southern Medical, Guangzhou 510515, Guangdong, China.; ^#^Authors contributed equally.

**Keywords:** Thyroid cancer, microRNA, autophagy, drug resistance, therapeutic targets

## Abstract

Thyroid cancer, particularly papillary thyroid cancer (PTC), represents the most prevalent endocrine malignancy. Despite advancements in therapeutic strategies, drug resistance significantly hampers clinical outcomes. Autophagy, an evolutionarily conserved cellular degradation pathway, acts paradoxically in thyroid cancer by promoting either tumor cell survival or cell death, thus influencing therapeutic resistance. Increasing evidence highlights microRNAs (miRNAs), small non-coding RNAs, as critical regulators of autophagy through precise modulation of autophagy-related genes (ATGs) and signaling pathways. miRNA-mediated autophagy can either enhance chemotherapeutic efficacy or facilitate resistance, depending on the cellular context and miRNA targets. This review summarizes recent insights into miRNA-autophagy interactions underlying drug resistance in thyroid cancer, emphasizing key miRNAs, including miR-125b, miR-144, miR-30d, and miR-9-5p. Understanding the complex regulatory networks connecting miRNAs and autophagy provides promising avenues for developing novel therapeutic strategies to overcome resistance in refractory thyroid cancer.

## INTRODUCTION

Thyroid cancer is the most common endocrine malignancy, with a steadily rising incidence worldwide over recent decades. It currently ranks as the ninth most common cancer worldwide, with women comprising approximately 75% of all cases. Among its subtypes, papillary thyroid cancer (PTC) is the most frequently diagnosed, accounting for nearly 80% of cases and typically associated with a favorable prognosis^[1,2]^.

Current standard therapies for thyroid cancer, especially differentiated thyroid cancer (DTC), include surgical resection, radioactive iodine (RAI) therapy, and thyroid-stimulating hormone suppression therapy^[3]^. In patients with radioiodine-refractory disease, targeted treatment with multi-kinase inhibitors, including lenvatinib and sorafenib, has demonstrated clinical efficacy^[4]^. In advanced cases, such as anaplastic thyroid cancer (ATC), recent therapeutic developments have focused on mutation-specific inhibitors - such as dabrafenib and trametinib for B-Raf proto-oncogene (BRAF)-mutated ATC - as well as immune checkpoint inhibitors, aiming to enhance clinical outcomes^[5]^. Despite the generally favorable prognosis of thyroid cancer, refractory cases remain a significant therapeutic challenge due to the emergence of treatment resistance^[6,7]^.

Due to the heterogeneity of thyroid cancer subtypes, diverse resistance mechanisms require subtype-specific therapeutic strategies. In medullary thyroid cancer (MTC) with rearranged during transfection (RET) mutations, resistance to RET inhibitors may arise from secondary RET alterations and activation of bypass signaling pathways^[8]^. In the context of immunotherapy, resistance to anti-PD-1 treatment has been attributed to M2 macrophage-derived extracellular vesicles (EVs), which suppress methyltransferase-like 3 (METTL3) and stabilize CD70 mRNA^[9]^. In ATC, resistance to doxorubicin (DOX) has been linked to signal transducer and activator of transcription 3 (STAT3)-mediated enhancement of cancer stem cell characteristics via INO80 regulation^[10]^. Furthermore, activation of the fibroblast growth factor receptor (FGFR) pathway has been associated with resistance to kinase inhibitors through the MAPK and PI3K/AKT signaling cascades^[11]^. In BRAF-mutant PTC, pericyte-driven activation of the thrombospondin-1/transforming growth factor beta 1 axis (TSP-1/TGFβ1 axis) contributes to resistance to vemurafenib and sorafenib^[12]^. Autophagy has also been implicated in resistance, sustaining mitochondrial respiration and thereby promoting tolerance to BRAFV600E inhibitors in thyroid cancer cells^[13]^.

Autophagy acts as a double-edged sword in cancer, aiding cell survival under stress while also promoting therapeutic resistance^[14]^. This conserved cellular process involves the sequestration of cytoplasmic components within membrane-bound autophagic vesicles, which subsequently fuse with lysosomes to facilitate the degradation and recycling of their contents, thereby maintaining cellular homeostasis^[15,16]^. The association between autophagy and drug resistance has been well documented across a range of malignancies, including lung, liver, breast, colorectal, ovarian, and esophageal cancers^[17-23]^.

**Figure 1:**
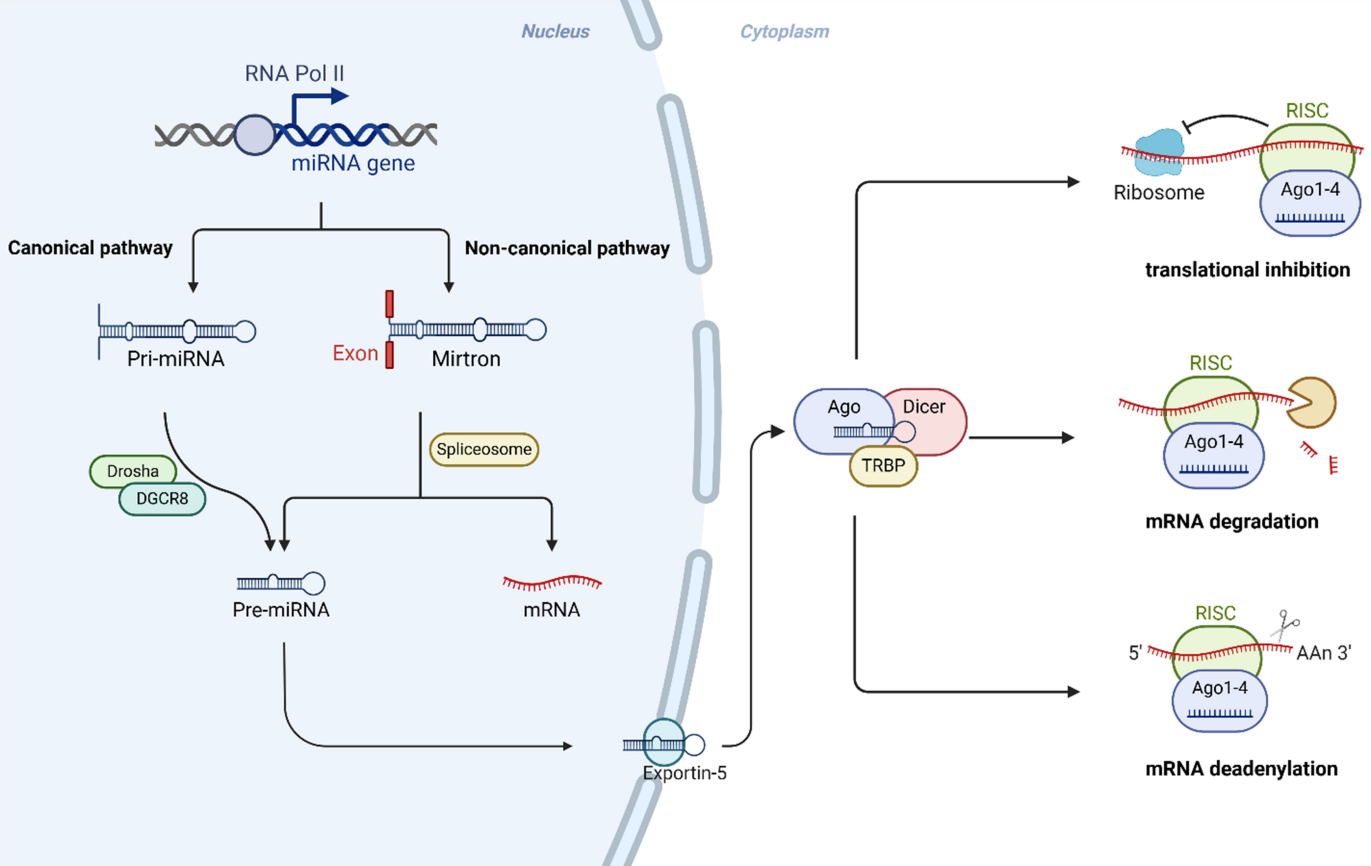
Schematic illustration of canonical and non-canonical miRNA biogenesis pathways and their mechanisms of action. In the canonical pathway, pri-miRNAs transcribed by RNA polymerase II undergo nuclear processing by the Drosha-DGCR8 complex into pre-miRNAs. Pre-miRNAs are exported to the cytoplasm via exportin-5 and further cleaved by Dicer to form mature miRNA duplexes. Mature miRNAs are incorporated into the RISC, guiding AGO proteins to their target mRNAs, leading to translational inhibition, mRNA degradation, or deadenylation. In contrast, the non-canonical pathway involves mirtrons - special intron-derived miRNAs - which bypass Drosha processing by utilizing host gene mRNA splicing machinery to generate pre-miRNAs directly. miRNA: MicroRNA; pri-miRNAs: primary miRNAs; DGCR8: DiGeorge syndrome critical region gene 8; pre-miRNAs: precursor miRNAs; RISC: RNA-induced silencing complex; AGO: Argonaute.

MicroRNAs (miRNAs) are endogenous, small non-coding RNAs approximately 21-23 nucleotides long, crucially regulating gene expression post-transcriptionally by repressing translation or triggering target mRNA degradation^[24-26]^. MiRNAs can be bound by long non-coding RNAs and circular RNAs within the competitive endogenous RNA network, thereby preventing targeted mRNA degradation^[27]^. The biogenesis of miRNAs is a tightly regulated multistep process involving enzymes such as Drosha, DiGeorge syndrome critical region gene 8 (DGCR8), exportin-5, Dicer, and Argonaute (AGO) proteins, where disruption in these pathways is associated with various pathologies, including cancers [Figure 1]. Dysregulation of miRNAs has been linked to altered cellular processes such as differentiation, proliferation, apoptosis, and metastasis^[25,28]^, and this dysregulation is associated with the progression and exacerbation of a variety of diseases^[29]^. Emerging evidence has underscored the pivotal role of miRNAs in regulating autophagy, with distinct miRNAs serving as positive or negative modulators of autophagy-related genes (ATGs)^[30]^. Increasingly, autophagy-driven drug resistance in cancer is linked to miRNA regulation, as specific miRNAs either enhance or inhibit autophagic pathways, significantly affecting tumor cell survival and therapy resistance^[31]^. The following section summarizes the documented interactions among miRNAs, autophagy, and drug resistance in thyroid cancer.

## miRNAs IN THYROID CANCER DRUG RESISTANCE

miRNAs have emerged as critical regulators of drug resistance across various malignancies, including thyroid cancer. These small non-coding RNA molecules modulate gene expression, thereby influencing the responsiveness of cancer cells to therapeutic interventions. Specifically, miRNAs regulate crucial cellular processes, such as apoptosis, proliferation, survival, and drug transport mechanisms, all intimately associated with therapy resistance. Through intricate regulatory networks, miRNAs participate in diverse cancer-related signaling pathways, providing insight into the underlying mechanisms driving treatment failure^[32]^. Indeed, miRNA-mediated regulation has been extensively investigated as a fundamental factor contributing to therapeutic resistance across multiple cancer types^[33]^. In the following sections, we summarize the relevant miRNAs reported in thyroid cancer drug resistance [Table 1]. These insights help elucidate the complex regulatory networks underpinning drug resistance and may inform future therapeutic strategies.

**Table 1:** The involved miRNAs in the drug resistance of thyroid cancer

**miRNA**	**Drug**	**Expression level**	**Target**	**Type of cancer**	**Ref.**
miR-27b-3p	DOX	Upregulated	PPARγ	ATC	[34]
miR-28-3p	DOX	Downregulated	BCL-2	PTC	[35]
miR-124-3p	Sorafenib	Downregulated	EZH2	DTC	[36]
miR-506-3p	Sorafenib	Downregulated	EZH2	DTC	[36]
miR-146	DOX and 5-Fu	Upregulated	KIT	PTC	[37]
miR-155	RAI	Upregulated	FOXO3	PTC	[38]
miR-206	Euthyrox	Downregulated	MAP4K3	PTC	[39]
miR-381-3p	DOX	Downregulated	HOXA9	ATC	[40]

miRNAs: MicroRNAs; DOX: doxorubicin; 5-Fu: 5-fluorouracil; RAI: radioactive iodine; PPARγ: peroxisome proliferator-activated receptor gamma; BCL-2: B cell lymphoma-2; EZH2: zeste homolog 2; KIT: KIT proto-oncogene, receptor tyrosine kinase; FOXO3: forkhead box O3; MAP4K3: mitogen-activated protein kinase kinase kinase kinase 3; HOXA9: homeobox A9; ATC: anaplastic thyroid cancer; PTC: papillary thyroid cancer; DTC: differentiated thyroid cancer.

In ATC, miR-27b-3p has been identified as a critical contributor to DOX resistance through its direct regulation of peroxisome proliferator-activated receptor gamma (PPARγ). This interaction is mediated by p65 NF-κB signaling, which induces the upregulation of miR-27b-3p, thereby reducing the sensitivity of tumor cells to chemotherapy-induced apoptosis. Conversely, silencing miR-27b-3p has been shown to effectively restore cellular sensitivity to DOX^[34]^. Similarly, miR-28-3p acts as an important regulator of chemotherapy resistance in PTC. Specifically, miR-28-3p targets the anti-apoptotic protein B cell lymphoma-2 (BCL-2); its suppression inhibits cellular proliferation and promotes apoptosis via modulation of the PI3K/AKT signaling pathway. Thus, the downregulation of miR-28-3p significantly enhances chemosensitivity and improves the response of PTC cells to DOX^[35]^. Moreover, reduced expression of miR-124-3p and miR-506-3p is implicated in resistance to sorafenib, a multi-kinase inhibitor frequently employed for advanced DTC treatment. Both miRNAs directly target enhancer of zeste homolog 2 (EZH2), a histone methyltransferase involved in epigenetic gene regulation; decreased expression of these miRNAs elevates EZH2 levels, leading to enhanced histone methylation and sorafenib resistance. Restoration of miR-124-3p and miR-506-3p expression has demonstrated efficacy in re-sensitizing thyroid cancer cells to sorafenib, highlighting their potential therapeutic^[36]^. Additionally, miR-146 exhibits significant upregulation in PTC and contributes to chemotherapy resistance by directly targeting the receptor tyrosine kinase KIT, thereby influencing tumor invasiveness and metastatic potential. This miRNA-mediated reduction of KIT expression correlates with diminished responsiveness to therapeutic agents^[37]^. Additionally, miR-155-mediated suppression of the long non-coding RNA cancer susceptibility candidate 2 (CASC2) has been associated with resistance to RAI therapy in thyroid cancer cells. Conversely, inhibition of miR-155, leading to CASC2 upregulation, enhances cellular sensitivity to RAI therapy^[38]^. Still, miR-206 has been reported to counteract resistance to euthyrox, a widely used thyroid hormone replacement therapy, by directly targeting mitogen-activated protein kinase kinase kinase kinase 3 (MAP4K3). This inhibition by miR-206 effectively suppresses the activation of the p38 and c-Jun NH2-terminal kinase (JNK) signaling pathways, key mediators of multidrug resistance. Consequently, elevated miR-206 levels significantly reduce euthyrox resistance in PTC cells^[39]^. Moreover, miR-381-3p sensitizes ATC cells to DOX by directly targeting the transcription factor homeobox A9 (HOXA9). Through this regulatory interaction, miR-381-3p inhibits cell proliferation and induces apoptosis, thereby reversing chemoresistance and highlighting its clinical potential for improving therapeutic responsiveness in aggressive thyroid cancers^[40]^.

In brief, miRNAs play significant roles in modulating drug resistance in thyroid cancer by regulating key molecular pathways associated with chemotherapy response. Further exploration of miRNA-mediated mechanisms, particularly those involving autophagy, holds substantial promise for advancing innovative strategies to overcome therapeutic resistance and enhance clinical outcomes in thyroid cancer.

## RELATIONSHIP BETWEEN AUTOPHAGY AND TUMOR DRUG RESISTANCE

Autophagy plays a dual role in tumor drug resistance: promoting tumor survival via protective autophagy^[41]^ or suppressing tumor progression through autophagy-induced cell death (AICD)^[42]^.

### Autophagy induces thyroid cancer drug resistance (cytoprotective autophagy)

As an adaptive cellular process, autophagy is induced by various external stimuli, including hypoxia, nutrient deprivation, and anticancer therapies, thereby functioning as a cytoprotective mechanism, called cytoprotective autophagy^[43]^.

In ATC, the AKT/mTOR signaling pathway has been identified as a central regulator of apatinib-induced cytoprotective autophagy, with autophagy inhibition markedly increasing apoptosis and improving therapeutic efficacy^[44]^. Similarly, treatment with BRAF inhibitors has been shown to activate protective autophagy via the AMP-activated protein kinase–Unc-51-like autophagy activating kinase 1 axis (AMPK–ULK1 axis), thereby contributing to drug resistance in BRAF-mutant thyroid cancers^[45]^. Cisplatin resistance has also been linked to miRNA-mediated modulation of autophagy, wherein specific miRNAs regulate the expression of ATGs, enabling tumor survival under chemotherapeutic stress^[46]^. In PTC, apatinib has been shown to induce autophagy through activation of the PI3K/AKT/mTOR pathway, and pharmacological inhibition of autophagy significantly enhances apoptotic cell death^[47]^. Moreover, in anlotinib-treated ATC, autophagic activation confers a survival benefit, whereas autophagy inhibition has been reported to potentiate ferroptosis, thereby improving antitumor responses^[48]^.

In a word, these findings highlight autophagy as a critical mediator of drug resistance in thyroid cancer and suggest that co-targeting autophagic pathways alongside standard therapies may enhance treatment efficacy and overcome resistance.

### AICD increases the sensitivity of thyroid cancer to drugs

In contrast to cytoprotective autophagy, which facilitates drug resistance, AICD promotes tumor elimination by disrupting cellular homeostasis and metabolic function. This dual role of autophagy presents a therapeutic opportunity for overcoming drug resistance in thyroid cancer^[49,50]^.

Recent investigations have underscored the potential of natural compounds in promoting autophagic cell death in thyroid malignancies. Berberine (BER) has been shown to induce both apoptosis and autophagy in ATC cells via inhibition of the PI3K/AKT/mTOR pathway, thereby enhancing sensitivity to DOX. Pharmacological blockade of autophagy with 3-methyladenine significantly attenuates BER-induced cytotoxicity, supporting a mechanistic role for autophagic cell death^[51]^. Similarly, allicin, a bioactive organosulfur compound derived from garlic, has been reported to potentiate the cytotoxic effects of cisplatin and carboplatin by inducing autophagy-dependent cell death in thyroid cancer cells, suggesting its utility as an adjunctive therapy^[52]^.

Curcumin, a polyphenolic compound derived from Curcuma longa, has been shown to induce mitophagy - a selective form of autophagy targeting damaged mitochondria - in PTC cells, thereby sensitizing them to radioiodine therapy. This effect is mediated through the upregulation of mitochondrial succinate dehydrogenase activity and enhanced production of reactive oxygen species (ROS), culminating in cell death^[53]^. In a separate study, curcumin was also found to induce autophagic cell death in thyroid cancer cells through activation of the MAPK pathway and concurrent inhibition of mTOR signaling, further supporting its therapeutic potential in overcoming drug resistance^[54]^. Aloperine, an alkaloid isolated from Sophora alopecuroides, has been shown to induce autophagic cell death in multidrug-resistant PTC and ATC cells through activation of the AMPK, ERK, JNK, p38, and AKT signaling pathways^[55]^. Reversine, a synthetic small molecule, similarly enhances drug sensitivity in follicular thyroid cancer (FTC) cells by suppressing the AKT/mTOR axis and promoting autophagic cell death^[56]^. Moreover, apigenin - a dietary flavonoid found in various fruits and vegetables - induces autophagy-dependent cell death in PTC cells via Beclin 1 accumulation, Cytosolic form of microtubule-associated protein 1A/1B-light chain 3 (LC3I) to phosphatidylethanolamine-conjugated form of microtubule-associated protein 1A/1B-light chain 3 (LC3II) conversion, and elevated ROS generation. This cascade results in DNA damage and G2/M phase cell cycle arrest, significantly enhancing chemotherapy sensitivity^[57]^.

In conclusion, these findings support the therapeutic promise of AICD as a strategy to counteract drug resistance in thyroid cancer. The modulation of autophagic pathways using natural compounds offers a novel and potentially effective approach to augment existing treatments and improve outcomes in therapy-resistant thyroid malignancies.

## EFFECTS OF miRNAs ON AUTOPHAGY-RELATED PROTEINS AND PATHWAYS INVOLVED IN THYROID CANCER DRUG RESISTANCE

**Figure 2:**
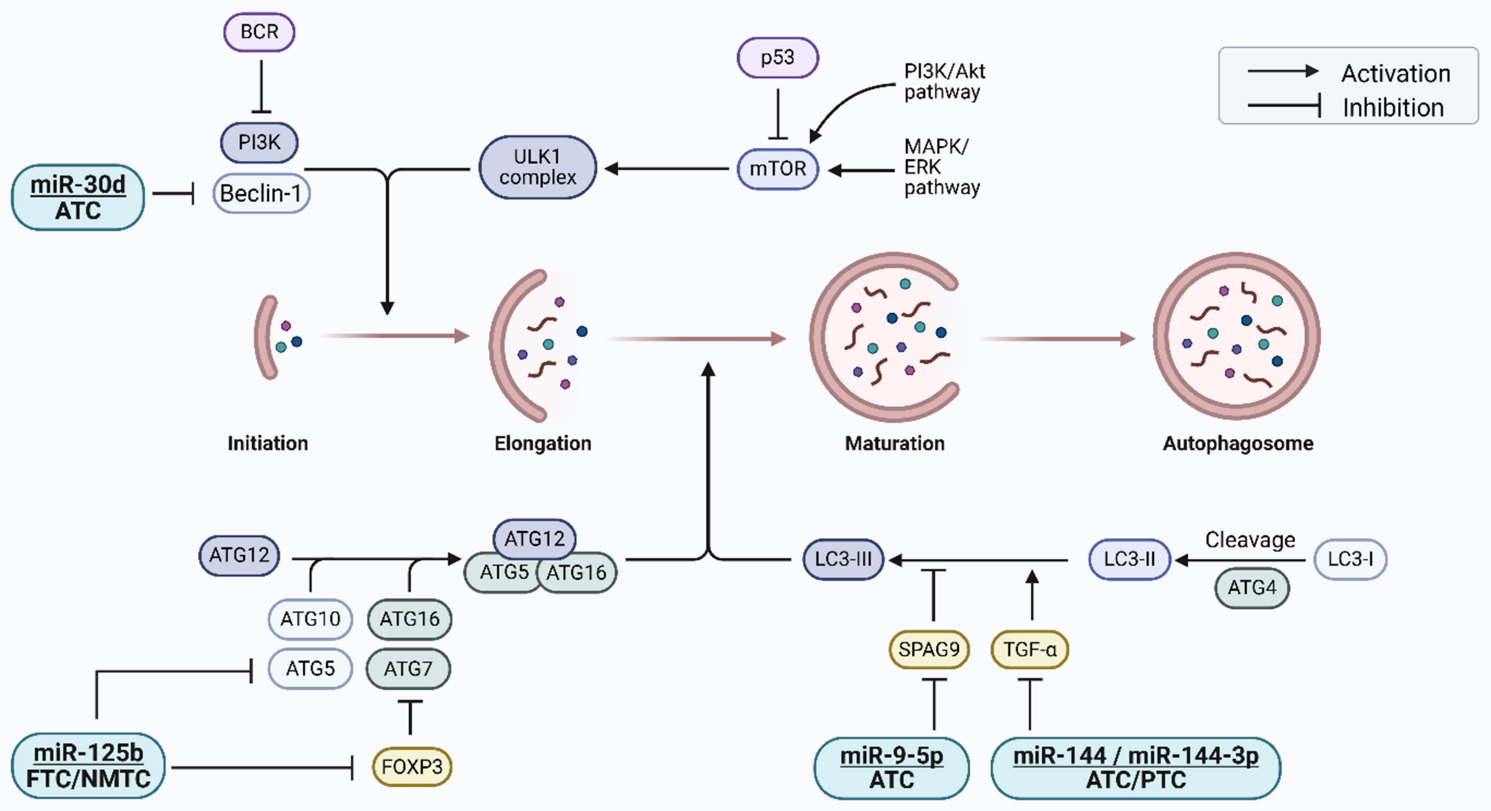
miRNAs and their targets involved in regulating autophagy and influencing drug sensitivity in thyroid cancer cells. miRNAs precisely modulate the expression of autophagy-related proteins, thus impacting drug resistance in thyroid cancer. miRNAs: MicroRNAs; BCR: B-cell receptor; PI3K: phosphoinositide 3-kinase; mTOR: mammalian target of rapamycin; ULK1 complex: unc-51 like autophagy activating kinase 1 complex; ATG: autophagy-related gene; Beclin-1: coiled-coil myosin-like BCL2-interacting protein; FOXP3: forkhead box protein P3; SPAG9: sperm-associated antigen 9; TGF-α: transforming growth factor-alpha; LC3I: cytosolic form of microtubule-associated protein 1A/1B-light chain 3; LC3II: phosphatidylethanolamine-conjugated form of microtubule-associated protein 1A/1B-light chain 3; FTC: follicular thyroid cancer; NMTC: non-medullary thyroid cancer; ATC: anaplastic thyroid cancer; PTC: papillary thyroid cancer.

In most previous studies, autophagy was modulated using pharmacological agents or chemical compounds that lacked target specificity. In contrast, miRNAs, by virtue of their mechanism of action, exert precise regulation over autophagy-related protein expression through base pairing with complementary mRNA sequences. This post-transcriptional regulation enables miRNAs to modulate autophagy with high specificity, thereby influencing drug resistance in thyroid cancer cells. miRNAs that affect drug sensitivity through this mechanism are summarized in Table 2 and Figure 2, with their regulatory relationships described in detail below.

**Table 2:** The involved miRNAs in autophagy regulation affecting drug sensitivity in thyroid cancer

**miRNA**	**Target**	**Autophagy protein**	**Autophagy**	**Sensitivity**	**Type of cancer**	**Ref.**
miR-125b	FOXP3	ATG7	↑	↑ (Cisplatin)	FTC	[58]
miR-125b	ATG5	ATG5	↓	↑ (Chemo and RAI)	NMTC	[59,60]
miR-144	TGF-α	LC3 II/LC3 I	↓	↑ (Cisplatin)	ATC	[61]
miR-144-3p	TGF-α	LC3 II/LC3 I	↓	↑ (Cisplatin)	PTC and ATC	[62]
miR-30d	Beclin 1	Beclin 1	↓	↑ (Cisplatin)	ATC	[63]
miR95p	SPAG9	LC3 II/LC3 I	↑	↑ (Cisplatin)	ATC	[64]

miRNAs: MicroRNAs; FOXP3: forkhead box protein p3; ATG: autophagy-related gene; TGF-α: transforming growth factor-alpha; Beclin-1: coiled-coil myosin-like BCL2-interacting protein; SPAG9: sperm-associated antigen 9; LC3I: cytosolic form of microtubule-associated protein 1A/1B-light chain 3; LC3II: phosphatidylethanolamine-conjugated form of microtubule-associated protein 1A/1B-light chain 3; Chemo: chemotherapeutic drug; RAI: radioactive iodine; FTC: follicular thyroid cancer; NMTC: non-medullary thyroid cancer; ATC: anaplastic thyroid cancer; PTC: papillary thyroid cancer.

### ATG proteins and miR-125b

Autophagy is an evolutionarily conserved, lysosome-dependent catabolic process that maintains cellular homeostasis through the degradation and recycling of damaged organelles, misfolded proteins, and cytoplasmic constituents. This complex mechanism is orchestrated by a network of ATG proteins acting in a coordinated sequence across key stages, including initiation, nucleation, elongation, maturation, and fusion of autophagosomes with lysosomes^[65]^. These ATG proteins are organized into core functional complexes - such as the ULK1/2 kinase complex, the autophagy-specific class III PI3K complex, the ATG9A trafficking system, and two ubiquitin-like conjugation systems (ATG12–ATG5–ATG16L and LC3) - that precisely regulate membrane dynamics required for autophagosome biogenesis^[66]^ and have been shown to be involved in the progression of malignant tumors^[67]^.

miR-125b has emerged as a critical regulator of autophagy-mediated drug resistance in FTC, acting through modulation of forkhead box protein P3 (FOXP3), ATG7, and ATG5. In FTC, reduced miR-125b expression results in the upregulation of FOXP3, which suppresses autophagy and contributes to cisplatin resistance. In contrast, elevated miR-125b levels inhibit FOXP3, thereby promoting ATG7 expression, enhancing autophagy, and sensitizing tumor cells to cisplatin treatment^[58]^. Beyond ATG7 regulation, miR-125b also directly targets ATG5, a central component of the autophagosome formation machinery. Downregulation of miR-125b leads to increased ATG5 expression, triggering excessive autophagic activity that promotes chemoresistance in non-medullary thyroid cancer (NMTC). This autophagy-driven survival mechanism allows tumor cells to escape apoptosis, particularly in the context of resistance to RAI therapy^[59,60]^.

Collectively, these findings indicate that miR-125b exerts a dual regulatory influence on autophagy-associated drug resistance by modulating both FOXP3-mediated ATG7 expression and ATG5-dependent autophagy. Targeting the miR-125b/ATG regulatory axis may offer a promising strategy to overcome autophagy-mediated therapeutic resistance and improve the efficacy of chemotherapy in thyroid cancer.

### TGF-α and miR-144

Transforming growth factor-alpha (TGF-α), a key ligand of the epidermal growth factor receptor (EGFR), plays a pivotal role in promoting drug resistance across multiple cancer types, including thyroid cancer. Acting through both autocrine and paracrine mechanisms, TGF-α activates EGFR-dependent signaling pathways - such as the Ras/Raf/MEK/ERK and PI3K/AKT/mTOR cascades - that support cancer cell proliferation, survival, and therapeutic resistance^[68]^. Tumor cells overexpressing TGF-α display both primary and acquired resistance to kinase inhibitors, including anaplastic lymphoma kinase (ALK) and mesenchymal-epithelial transition factor (c-Met) inhibitors, by maintaining pro-survival signaling and inhibiting apoptosis, underscoring its role as a central mediator of drug resistance^[69]^. Furthermore, TGF-α contributes to intercellular crosstalk within heterogeneous tumor cell populations, facilitating the emergence of more aggressive and drug-resistant clones through paracrine signaling, thereby complicating therapeutic efficacy^[70]^. Given its association with tumor progression and resistance, targeting TGF-α-driven pathways represents a promising therapeutic strategy for improving drug response in thyroid cancer.

Mounting evidence indicates that miR-144 plays a key role in regulating autophagy and drug resistance in thyroid cancer by directly targeting TGF-α. In ATC, a highly aggressive subtype, miR-144 expression is markedly reduced, whereas TGF-α expression is elevated, contributing to enhanced tumor progression and cisplatin resistance. Functional studies demonstrate that restoration of miR-144 sensitizes ATC cells to cisplatin by suppressing autophagy, a survival mechanism frequently exploited by cancer cells under chemotherapeutic stress^[61]^. Moreover, miR-144-3p, a principal isoform of miR-144, has been identified as a negative regulator of TGF-α-mediated autophagy and chemoresistance in both PTC and ATC. Recent findings reveal that circEIF6, a circular RNA, functions as a competitive endogenous RNA that sponges miR-144-3p, preventing it from targeting TGF-α. Depletion of circEIF6 results in increased miR-144-3p levels, leading to downregulation of TGF-α, inhibition of autophagy, and heightened sensitivity to cisplatin in thyroid cancer cells. Conversely, circEIF6 overexpression elevates TGF-α levels, enhancing autophagy, reducing apoptosis, and promoting cisplatin resistance^[62]^.

Together, these studies underscore the miR-144/miR-144-3p–TGF-α regulatory axis as a pivotal modulator of autophagy-driven drug resistance in thyroid cancer. Targeting this pathway - either by restoring miR-144-3p function or inhibiting TGF-α signaling - represents a promising therapeutic strategy for reversing cisplatin resistance and enhancing treatment efficacy.

### Beclin 1 and miR-30d

Beclin 1 is a central regulator of autophagy and plays a crucial role in modulating drug resistance in various cancers, including thyroid cancer. As a key component of the class III phosphatidylinositol 3-kinase (PI3KC3) complex, Beclin 1 regulates the initiation of autophagosome formation, which is essential for maintaining cellular homeostasis under stress conditions^[71]^. However, its role in cancer is complex - while Beclin 1-mediated autophagy can act as a tumor suppressor by preventing genomic instability, excessive autophagy can promote tumor survival by enhancing chemoresistance. In thyroid cancer, studies have shown that Beclin 1 expression is upregulated in response to cisplatin treatment, contributing to autophagy-dependent drug resistance. Mechanistically, Beclin 1 interacts with key autophagy-related proteins, including ATG5 and ATG7, to facilitate the formation and maturation of autophagosomes, thereby sustaining cancer cell survival during chemotherapy. These findings suggest that therapeutic targeting of Beclin 1-mediated autophagy may offer a promising approach to sensitizing thyroid cancer cells to anticancer treatments^[72]^.

miRNAs have been identified as post-transcriptional regulators of Beclin 1, among which miR-30d has gained attention for its role in modulating autophagy and drug resistance in thyroid cancer. In ATC, a highly aggressive subtype, miR-30d, is significantly downregulated, leading to enhanced Beclin 1-mediated autophagy and reduced sensitivity to cisplatin. Functional studies have confirmed that miR-30d directly targets the 3′-untranslated region (3′-UTR) of Beclin 1 mRNA, resulting in its degradation and subsequent inhibition of autophagy. Restoration of miR-30d expression suppresses autophagy, enhances apoptosis, and sensitizes ATC cells to cisplatin treatment in both *in vitro* and *in vivo* models. These findings highlight the therapeutic potential of miR-30d in overcoming Beclin 1-mediated chemoresistance^[63]^.

The Beclin 1/miR-30d regulatory axis plays a pivotal role in modulating the balance between cytoprotective autophagy and apoptosis, thereby critically influencing chemoresistance in thyroid cancer. Considering autophagy’s context-dependent role in tumor progression, restoring miR-30d to suppress Beclin 1-mediated autophagy is a promising approach to enhance chemotherapy effectiveness and overcome resistance in thyroid cancer.

### SPAG9 and miR-9-5p

Sperm-associated antigen 9 (SPAG9), a recently characterized cancer-testis antigen, is a member of the JNK-interacting protein family and shares structural homology with JNK-interacting protein 3 (JIP3). Initially identified in human testicular tissue, SPAG9 specifically localizes within the sperm acrosomal compartment, where it facilitates sperm-egg interactions^[73]^. Notably, accumulating evidence has linked SPAG9 to increased tumor invasiveness, metastatic potential, chemoresistance, and unfavorable prognosis across multiple malignancies, including gastric cancer, hepatocellular cancer, renal cell cancer, prostate cancer, and osteosarcoma^[74-78]^.

In ATC, miR-9-5p has emerged as a crucial regulator of autophagy-associated chemoresistance via direct modulation of SPAG9 expression. Specifically, miR-9-5p negatively regulates SPAG9 by interacting with complementary sequences within its 3′-UTR. Decreased miR-9-5p levels result in elevated SPAG9 expression, subsequently enhancing autophagic flux, as indicated by an increased LC3II/I ratio. This heightened autophagic activity mediates resistance of ATC cells to treatment. Conversely, restoration of miR-9-5p expression significantly attenuates SPAG9 levels, reduces the LC3II/I ratio, inhibits autophagy, and thereby restores chemosensitivity, facilitating apoptosis in response to chemotherapy^[64]^.

In summary, these findings underscore the miR-9-5p/SPAG9 regulatory axis as a pivotal mechanism underlying autophagy-associated drug resistance in thyroid cancer. Further exploration of this molecular pathway may offer novel therapeutic targets to overcome resistance and improve clinical efficacy in treating refractory thyroid cancer.

## CONCLUSIONS AND PERSPECTIVES

MiRNA-mediated autophagy plays a critical role in thyroid cancer drug resistance, influencing both tumor survival and therapy sensitivity. While extensively studied in other cancers, its role in thyroid cancer remains underexplored. In addition to miRNA-mediated regulation of autophagy, EVs have emerged as critical players in cancer drug resistance^[79,80]^. Understanding the interplay between EVs and miRNA-mediated autophagy could offer new avenues for overcoming drug resistance in thyroid cancer^[81,82]^.

Autophagy plays a dual role in thyroid cancer drug resistance, acting as either a cytoprotective mechanism that sustains tumor survival or a cell death pathway that enhances therapy sensitivity. miRNAs have emerged as key regulators of ATG, modulating autophagic activity and thereby influencing cancer progression and drug response. The interplay between miRNAs and autophagy ultimately determines whether thyroid cancer cells evade apoptosis or succumb to therapy-induced cell death. Cytoprotective autophagy, often triggered by therapy, facilitates tumor adaptation to chemotherapeutic stress, leading to drug resistance. In contrast, AICD represents a therapeutic vulnerability that can be exploited to enhance drug efficacy.

Autophagy, regulated by miRNAs, plays a pivotal role in drug resistance across multiple cancer types. In cancers such as breast cancer, osteosarcoma, and lung cancer, extensive research has demonstrated that miRNA-mediated autophagy critically influences drug response. In breast cancer, miRNAs serve as key modulators of autophagy, impacting treatment resistance. Research highlights that miR-125b downregulation increases ATG5 and ATG7 expression, thereby enhancing autophagy and promoting resistance to chemotherapeutic agents. This mechanism of action is similar to that of miR-125b in FTC and NMTC, but has different clinical outcomes^[58-60]^. Similarly, miR-221/222 overexpression induces autophagy-mediated resistance in tamoxifen-treated breast cancer cells, while suppression of these miRNAs restores sensitivity to treatment^[83]^. In osteosarcoma, miRNA-mediated autophagy has been strongly implicated in drug resistance. Studies report that miR-101 inhibits autophagy by targeting ATG4D, thereby sensitizing osteosarcoma cells to cisplatin. Another study demonstrates that miR-30a directly suppresses Beclin-1, reducing autophagic activity and reversing resistance to DOX, which is the same mechanism of action as miR-30d in ATC and has the same clinical results^[63]^. Additionally, miR-199a-5p downregulation is associated with enhanced autophagic flux, contributing to poor chemotherapeutic response^[84]^. Lung cancer research further underscores the importance of miRNA-mediated autophagy in therapeutic resistance. Findings show that miR-200c suppresses autophagy by targeting ULK1, thereby increasing chemosensitivity in non-small cell lung cancer (NSCLC). Additionally, miR-223 has been implicated in promoting resistance through enhanced autophagic flux, while miR-26b restoration, coupled with autophagy suppression, reduces cisplatin resistance^[85]^. Broader analyses across multiple cancers indicate that miRNA-mediated autophagy is a well-documented resistance mechanism. Reviews on miRNA-autophagy interactions in chemotherapy resistance highlight significant roles in various tumors, such as colorectal and pancreatic cancers^[86]^. The cumulative evidence suggests that modulating miRNA expression could be a promising strategy to overcome therapy resistance, and these findings suggest the existence of a potential miRNA regulatory axis that warrants further validation in thyroid cancer models. However, while extensive studies have established miRNA-autophagy interactions as key determinants of drug resistance in various cancers, thyroid cancer research in this area remains limited. Research on miRNA-mediated regulation of autophagy remains particularly limited within the pathological classifications of MTC and FTC. While miR-183^[87]^ and miR-9-3p^[88]^ have been implicated in modulating autophagy and influencing tumor biology in MTC, offering potential therapeutic targets, their roles in treatment resistance remain insufficiently explored. Similarly, investigations into miRNA-mediated regulation of autophagy in FTC remain limited, with a notable paucity of data. Although some studies suggest that miRNAs regulate autophagy in thyroid cancer, the available evidence is not as comprehensive or well-characterized as in other malignancies. This highlights the need for further investigation to determine whether targeting miRNA-autophagy pathways could provide therapeutic benefits in thyroid cancer.

Given the established role of miRNA-mediated autophagy in cancer drug resistance, therapeutic strategies targeting this axis are gaining traction. In breast cancer, miRNA-based interventions have been proposed as a means to enhance chemotherapy efficacy. For instance, restoring miR-34a levels suppresses autophagy and increases sensitivity to DOX. Similar approaches involving miR-124 and miR-200c have also been explored to counteract drug resistance^[83]^.

Autophagy inhibitors represent another promising avenue for therapy. Studies in osteosarcoma have shown that pharmacological inhibition of autophagy using chloroquine (CQ) or hydroxychloroquine (HCQ) sensitizes tumor cells to chemotherapy. Additionally, dual inhibition strategies targeting both autophagy and key signaling pathways, such as PI3K/AKT/mTOR, have demonstrated enhanced drug efficacy^[84]^. In lung cancer, preclinical studies have explored combining autophagy inhibitors with conventional therapies. Research reports that using miRNA mimics alongside autophagy inhibitors significantly enhances the efficacy of chemotherapy. Specifically, the combination of miR-26b restoration and autophagy suppression reduces cisplatin resistance in lung adenocarcinoma cells^[85]^. Promising clinical efficacy has been demonstrated for CQ or HCQ in combination with chemotherapy in multiple clinical trials. In a phase II trial, combining CQ (250 mg/day) with taxane-based chemotherapy in anthracycline-refractory breast cancer resulted in an overall response rate (ORR) of 45.2%, significantly surpassing the anticipated 30%, alongside median progression-free survival (PFS) of 12.4 months and overall survival (OS) of 25.4 months, highlighting both clinical effectiveness and manageable toxicity^[89]^. Similarly, a phase Ib/II trial evaluating HCQ (200 mg twice daily) in combination with carboplatin/paclitaxel (and bevacizumab if criteria met) in metastatic NSCLC demonstrated an ORR of 33% and a disease control rate of 53%, further supporting autophagy inhibition as a viable approach to enhance chemotherapy outcomes^[90]^. Recent reviews on cancer chemoresistance further emphasize the potential of miRNA-based therapies^[86]^.

The evidence mentioned above reveals that miRNA-mediated regulation of autophagy consistently contributes to drug resistance across various therapeutic classes, including cytotoxic chemotherapy, targeted kinase inhibitors, and radioiodine therapy, in thyroid cancer, involving core autophagy-related proteins such as ATG5, ATG7, Beclin 1, and SPAG9. Commonly, reduced expression of specific miRNAs, including miR-125b, miR-144-3p, miR-30d, and miR-9-5p, enhances autophagic flux and thereby facilitates tumor cell survival under chemotherapeutic stress. Despite these shared regulatory targets, distinct miRNA-protein interactions such as FOXP3 with ATG7 or ATG5, TGF-α, Beclin 1, and SPAG9 function specifically within different cancer subtypes, including FTC, NMTC, and ATC, underscoring the necessity for subtype-specific therapeutic strategies.

Although targeting autophagy in thyroid cancer treatment has considerable potential, it is hampered by multiple challenges. Primarily, autophagy exhibits a paradoxical duality: it functions as a cytoprotective mechanism that enables cancer cells to survive therapeutic stress, while under other conditions, it also mediates AICD^[91]^. This intricate dual role complicates the strategic modulation of autophagy, necessitating precise delineation of cellular contexts to predict therapeutic outcomes effectively. Additionally, the clinical translation of autophagy-targeted therapies, including lysosomotropic inhibitors such as CQ and HCQ, is constrained by significant toxicity profiles^[92]^, thereby restricting their therapeutic window, limiting dose intensity, and impeding long-term clinical application. Moreover, miRNA-based therapies aimed at regulating autophagy have also encountered significant obstacles, such as inefficient and unstable delivery systems; although ongoing advancements in delivery technology and molecular design strategies seek to mitigate these limitations, such challenges continue to complicate the clinical translation of miRNA therapeutics^[93,94]^. Therefore, overcoming these challenges through detailed mechanistic insights, improved delivery strategies for miRNA-based therapies, and precise molecular characterization will be essential for optimizing autophagy modulation strategies in thyroid cancer therapy. Recent studies have suggested that autophagy may play a pivotal role in immune escape through the modulation of key processes such as antigen presentation, cytokine secretion, and immune checkpoint expression^[95,96]^. Considering the regulatory function of miRNAs in autophagy, it is conceivable that miRNA-mediated autophagy could significantly influence immune evasion in thyroid cancer. Exploring this intricate relationship could reveal novel mechanisms of resistance and contribute to the refinement of immunotherapeutic strategies.

Studies have also shown that miRNA-mediated autophagy modulation can be exploited to counteract resistance in multiple tumor types^[31]^. Although such approaches have been widely studied in gastrointestinal and hematological cancers, their application in thyroid cancer remains largely unexplored. With expanding research into miRNA-mediated autophagy, novel therapeutic opportunities are arising to combat drug resistance in thyroid cancer. Additionally, preclinical and clinical investigations combining miRNA-based therapies with autophagy inhibitors could provide a more effective strategy for enhancing treatment response. Given the extensive evidence supporting miRNA-autophagy interactions in other malignancies, further exploration in thyroid cancer may lead to novel, precision-targeted interventions that improve patient outcomes. Future research should focus on identifying thyroid cancer-specific miRNAs and integrating autophagy modulation with existing treatments. Advancing our understanding of these mechanisms could lead to precision-targeted therapies, improving drug efficacy and patient outcomes in refractory thyroid cancer.

## DECLARATIONS

### Copyright

© The Author(s) 2025.
